# Genomics of Plant Disease Resistance in Legumes

**DOI:** 10.3389/fpls.2019.01345

**Published:** 2019-10-30

**Authors:** Prasanna Kankanala, Raja Sekhar Nandety, Kirankumar S. Mysore

**Affiliations:** Noble Research Institute, LLC, Ardmore, OK, United States

**Keywords:** genomics, legumes, plant–pathogen interactions, transcriptome analysis, GWAS, QTLs, markers, CRISPR/Cas9

## Abstract

The constant interactions between plants and pathogens in the environment and the resulting outcomes are of significant importance for agriculture and agricultural scientists. Disease resistance genes in plant cultivars can break down in the field due to the evolution of pathogens under high selection pressure. Thus, the protection of crop plants against pathogens is a continuous arms race. Like any other type of crop plant, legumes are susceptible to many pathogens. The dawn of the genomic era, in which high-throughput and cost-effective genomic tools have become available, has revolutionized our understanding of the complex interactions between legumes and pathogens. Genomic tools have enabled a global view of transcriptome changes during these interactions, from which several key players in both the resistant and susceptible interactions have been identified. This review summarizes some of the large-scale genomic studies that have clarified the host transcriptional changes during interactions between legumes and their plant pathogens while highlighting some of the molecular breeding tools that are available to introgress the traits into breeding programs. These studies provide valuable insights into the molecular basis of different levels of host defenses in resistant and susceptible interactions.

## Introduction

Legumes belong to the third-largest angiosperm family, Fabaceae or Leguminosae. This family comprises around 750 genera and 20,000 species, including grain, forage, and economically important legumes (Polhill et al., 1981). Legumes contribute 33% of human dietary protein (Vance et al., 2000). Although legumes are cultivated over 12 to 15% of the Earth’s arable land and account for 27% of the world’s primary crop production (Vance et al., 2000), their yield is limited due to environmental adaptability challenges and damage caused by pests and pathogens (Graham and Vance, 2003). Some of the major fungal diseases of legumes include rusts, mildews, root rots, wilts, blights, and anthracnoses. Bacterial diseases are mainly grouped into leaf blights, leaf spots, bacterial wilts, and a diverse group with symptoms such as dwarfing and rots (Rubiales et al., 2015; Wille et al., 2019). Viral diseases are caused by *Bean pod mottle virus, Soybean mosaic virus, and Peanut stripe virus*, among others. Cyst and root-knot nematodes are the devastating parasites of legumes (Rubiales et al., 2015).

Plants have evolved robust defense mechanisms against pathogen attack that are triggered by initial recognition of the pathogen. These mechanisms involve a cascade of signaling responses known as Pathogen-Associated Molecular Pattern (PAMP) Triggered Immune (PTI) response, which eventually leads to changes in the gene expression of the host. Depending on the type of interaction, this can result in either disease susceptibility or disease resistance. The pathogens, on the other end of the spectrum, have evolved several mechanisms involving effector delivery to evade the host defenses. The host defense response to effectors is called Effector-Triggered Immunity (ETI) (Young et al., 2005). The continuous arms race between the host and pathogen eventually determines the outcome of the interaction (Jones and Dangl, 2006). The host responses also vary based on pathogen infection strategies. The current understanding is that successful defense responses against biotrophic pathogens are predominantly mediated by the salicylic acid (SA)-dependent pathway and that those against hemibiotrophs and necrotrophs involve ethylene and jasmonic acid (JA) signaling (Glazebrook, 2005).

Leguminosae includes a diverse variety of plants. *Medicago truncatula* and *Lotus japonicus* have been chosen as model species to advance the study of legumes (Zhu et al., 2005). Several genetic and genomic resources have been developed in these two model legumes to assist breeding programs for enhanced tolerance/resistance to abiotic and biotic stresses in legume crop species. These include genome sequences (Sato et al., 2008; Young et al., 2011), expressed sequence tags (ESTs) (Asamizu et al., 2004; Gamas et al., 2006), physical and genetic maps (Choi et al., 2004; Yan et al., 2004; Young et al., 2005; Wang et al., 2008; Ohmido et al., 2010; Shah et al., 2016), and insertional mutagenesis lines (Tadege et al., 2008; Urbanski et al., 2013), among others. In addition to the model plants, the genome sequences of crop plants such as *Glycine max* (cultivated soybean), *Glycine soja* (wild soybean), *Cajanus Cajun* (pigeon pea), *Cicer arietinum* (chickpea), *Vigna radiata* (mung bean), *V. angularis* (adzuki bean), *V. unguiculata* (cowpea), *Arachis hypogaea* (cultivated peanut), *A. duranensis* (wild peanut A genome), *A. ipaensis* (wild peanut B genome), *Medicago sativa* (alfalfa), *Phaseolus vulgaris* (common bean), *Trifolium pretense* (red clover), *Lupinus angustifolius* (lupin), and *Lens culinaris* (lentil) are currently available at https://legumeinfo.org/genomes. The macrosynteny and microsynteny studies among some of these genomes have been useful for translating the knowledge from model to crop plants (Zhu et al., 2005). The availability of genome sequences coupled with recent advancements in affordable Next-Generation Sequencing (NGS) techniques and bioinformatics tools has enabled extensive study of genome-wide expression changes during plant–pathogen interactions to identify the pathways involved in plant defense. Macroarrays, microarrays, RNAseq, suppressive subtractive hybridization (Mehrtens et al., 2005), cDNA-amplified fragment length polymorphism (AFLP) techniques and gene-expression atlases have been used extensively to identify candidate genes for disease resistance. In this review, we focus on the interactions of legumes with plant pathogens such as fungi, oomycete, bacteria, nematodes, and viruses at the genomic level and the use of genomic technologies in breeding for resistance.

## Using Genomics to Understand the Basics of Plant–Pathogen Interactions in Legumes

### Genomics of Plant–Fungal Interactions

Fungi are among the most challenging plant pathogens to tackle owing to their genetic flexibility and plasticity, which allow them to adapt quickly to their changing environments (Perez-Nadales et al., 2014). Considerable effort has gone into understanding the plant–fungal interaction mechanisms in both model and crop legumes. Large-scale genomic studies have enabled understanding of the various plant disease-resistance mechanisms against hemibiotrophic, biotrophic, and necrotrophic fungal pathogens.

### Hemibiotrophic Interactions

*Mycosphaerella pinodes* is a broad-host range fungal pathogen that causes ascochyta blight disease. It is known to have a transient biotrophic phase in some hosts and to behave like a necrotrophic pathogen in other hosts (Fondevilla et al., 2011; Almeida et al., 2015). *M. truncatula*-based microarrays were used to study resistant interactions of pea with *M. pinodes*. The functional gene categories involved in the resistance mechanism included phytohormones, *Pathogenesis Related* (*PR*) genes, the phenylpropanoid pathway, cell-wall fortification, and genes involved in ethylene- and jasmonic-acid(JA)-related defense pathways (Fondevilla et al., 2011). This work was later augmented by investigating the transcriptome in the host pea plants using deep SuperSAGE analysis to enrich for transcripts in the pea–*M. pinodes* interactions, followed by next-generation sequencing (NGS) of the transcripts (Fondevilla et al., 2014). Several factors that play key roles in resistance were identified, such as the WRKY protein in pathogen perception, proteases as an active defense against fungal toxins, and the roles of ethylene, abscisic acid, and indole-3-acetic acid as phytohormones in defense. Flavonoids, terpenoids, reactive oxygen species (ROS) and phytoalexins were identified as antifungal components that inhibit hyphal growth and destroy toxins (Fondevilla et al., 2014).

In *Vigna unguiculata* (cowpea) and orphan legumes such as *Lathyrus sativus* (grass pea) and *Vicia faba* (fava bean) where whole-genome sequence information is not available, SuperSAGE and Deep SuperSAGE coupled with NGS sequencing has been used successfully to study transcriptomes during *Ascochyta* infections (Madrid et al., 2013; Almeida et al., 2015). Early gene expression profiling in a resistant variety of grass pea during *Ascochyta* infection identified that classical defense-response genes involved in cell-wall fortification and the phenylpopanoid pathway were differentially expressed in resistant interactions. In addition, homologs of several candidate resistance genes such as receptor kinases containing thaumatin-like protein (TLP) domains, leucine-rich repeat (LRR) domains, and a gene homologous to Resistance to *Pseudomonas syringae* pv *maculicola* 1 (*RPM1*) involved in conferring resistance to *P. syringae* in Arabidopsis were identified (Almeida et al., 2015). During resistant interactions in the fava bean-*Ascochyta fabae* infection process, genes involved in JA signaling and pectin esterase-encoding genes were identified with the SuperSAGE technique (Madrid et al., 2013). In a later study, *de novo* transcriptome assembly was used to identify transcripts in susceptible and resistant interactions of fava bean and *A. fabae*. Genes encoding LRR proteins, Rho2 GTPase-activating protein 2 (RGA2), several plant growth regulators, heat shock proteins, chitin elicitor-binding protein, and those genes that produce chlorogenic acid, scopoletin, and flavonoids were among the significant genes involved in fava bean defenses (Ocaña et al., 2015). Functional characterization of these candidate genes will be the next essential step toward including them in breeding programs.

Anthracnose disease is caused by a hemibiotrophic fungal pathogen, *Colletotrichum* spp. The genomics of *Colletotrichum*–host and –nonhost interactions were investigated using microarray analysis in *M. truncatula* (Jaulneau et al., 2010). In this study, resistant and susceptible *M. truncatula* varieties were infected with pathogenic strain *Colletotrichum trifolii* and non-adapted strains *C. higginsianum* and *C. lindemuthianum*. Resistance responses to non-adapted *Colletotrichum* spp. were similar to the incompatible responses induced by the adapted strain on the resistant line. The nonhost responses included localized oxidative burst and fluorescent compound release. The host resistance response was characterized through defense gene signaling and SA accumulation (Jaulneau et al., 2010). To identify the genetic components of bean immunity against *C. lindemuthianum*, EST analysis was carried out in common bean with putative *A. thaliana* orthologs (Oblessuc et al., 2012). This study suggested that ETI-triggered hypersensitive response is mediated by downregulation of *FLS2*-like and *MKK-5* like putative orthologs of *A. thaliana* genes involved in pathogen perception (Oblessuc et al., 2012). The resistant and susceptible interactions of *P. vulgaris* with *C. lindemuthianum* were investigated using NGS methods (Padder et al., 2016). Most of the DEGs were expressed in the biotrophic phase in the susceptible interaction, while most of the DEGs were expressed in the necrotrophic phase in the resistant interaction. DEGs in the resistant interaction were over-represented by genes expressing PR proteins and peroxidases, while the susceptible interaction was over-represented by genes encoding sugar transporters (Padder et al., 2016). The genomics of partially resistant and susceptible interactions of *Lens culinaris* and *C. lentis* were studied using EST analysis (Bhadauria et al., 2017). Twenty-six resistance genes were identified during the symptomatic phase of infection in the compatible interaction. Further, a complex interplay of plant hormone pathways was also observed in this study (Bhadauria et al., 2017).

Fusarium wilt is a destructive disease in several legumes that is caused by host-specific *Fusarium oxysporum* strains. Extensive studies have been done to understand the molecular basis of this disease interaction in various legumes. In *Phaseolus vulgaris* (common bean), the cDNA-AFLP technique was used to determine transcriptionally regulated genes in response to *F. oxysporum* f. sp. *phaseoli* (*Fop*) infection in resistant and susceptible interactions (Xue et al., 2015). This study identified 122 defense-related gene fragments that are distributed across the genome. This distribution could serve to tag defense-related molecular markers in breeding programs (Xue et al., 2015). RNAseq analysis of *Glycine max* infected with both pathogenic and non-pathogenic strains of *F. oxysporum* identified over-representation of defense-related genes corresponding to necrosis in resistant interactions (Lanubile et al., 2015). RNAseq was carried out to understand the molecular differences in defense responses between cultivated and wild species of *Glycine max* against the pathogenic *F. oxysporum* Schltdl (Chang et al., 2019). That study identified the role of secondary metabolites and plant hormones in wild-type germplasm that could be adapted into cultivated species for enhanced resistance.

Several races of *F. oxysporum* f. sp. *Ciceri* (*Foc*) have been identified across the chickpea-growing regions of the world. While most *F. oxysporum* strains are considered as necrotrophic or hemibiotrophic pathogens, *Foc* race 1 (*Foc*1) is reported to be an obligate biotrophic pathogen of chickpea (Gupta et al., 2009). cDNA-AFLP-based analyses, cDNA-based microarrays, and cDNA RAPD methods have been used to study chickpea interactions with *Foc*1 (Nimbalkar et al., 2006; Ashraf et al., 2009; Gupta et al., 2009; Gupta et al., 2010; Gurjar et al., 2012). Although host responses during biotrophic infections are mediated by SA-dependent pathways, gene expression analyses in the above-referenced studies indicate non-traditional responses. The cDNA-AFLP method identified that genes encoding sucrose synthases, invertases, and β-amylase were induced in resistant interactions. The *14-3-3* gene expression was overrepresented in the susceptible interaction, indicating potential nutrient starvation, and the resistant interaction potentially copes with this sugar starvation by over-inducing sugar-metabolism genes. This study was highly suggestive that sugar also acts as a signaling molecule in response to pathogen perception (Gupta et al., 2010). A comparative study of resistant and susceptible interactions with *Foc* races 1, 2, and 7 was conducted in chickpea using the cDNA-RAPD method (Gurjar et al., 2012). This study identified a role for plant glucosyltransferase genes in resistance response. Further, race-dependent defense responses were observed (Gurjar et al., 2012). A similar study with resistant and susceptible interactions with *Foc* race 1, 2, and 4 was conducted recently with the LongSAGE method (Upasani et al., 2017). Clustering analysis and interaction networks of differentially expressed genes (DEGs) identified that the resistant interaction is characterized by ROS production, SA production, lignification, *R*-gene expression, and hormone homeostasis. The susceptible interaction was enriched for actin depolymerization genes, aquaporin genes, and tetrapyrrole synthesis genes (Upasani et al., 2017). Another study detailing the transcriptional changes during resistant and susceptible chickpea interactions with *Foc*1 was done using RNAseq analysis (Gupta et al., 2017). Plant pathogen interaction networks constructed with this transcriptional data identified several nodal hub genes that modulate defense responses and could be further characterized for resistance (Gupta et al., 2017). A microarray-based study of resistant and susceptible chickpea interaction transcriptomes with *Foc*1 was used to create regulatory gene networks (Ashraf et al., 2018). This study identified 76 disease- and immunity-related genes. The gene regulatory networks identified transcriptional plasticity in immune pathways and disease pathways during wilt interactions. This work also highlighted that the primary metabolic components are shared between defense and disease (Ashraf et al., 2018).

### Biotrophic Interactions

Asian soybean rust (ASR), caused by *Phakospora pachyrhizi*, is a devastating disease that is listed among the top five biotic threats to agriculture (Pennisi, 2010). Although six resistance genes, *Resistance to Phakospora pachyrhizi* (*Rpp1-6*), have been identified in soybean that confer resistance to ASR in a race-specific manner, no single soybean genotype can confer resistance to all races of the rust fungus (Langenbach et al., 2016a). Several studies have been conducted to identify key players in resistance to ASR. Initial studies to identify key players in the *R*-gene-mediated response of ASR employed the SSH complementary DNA (cDNA) method (Choi et al., 2008; Soria-Guerra et al., 2010b). These studies indicated a time-dependent coordinated gene expression pattern in *Rpp*-mediated resistant and susceptible interactions and identified the role of peroxidases and lipoxygenases in resistance. Later work using whole-genome microarrays confirmed these two findings and provided further detail (Van De Mortel et al., 2007; Panthee et al., 2009; Soria-Guerra et al., 2010a; Soria-Guerra et al., 2010b; Morales et al., 2013). Several of these studies have reported an overrepresentation of transcription factors (TF) and the roles of flavonoids and cell-wall lignification in the active resistance mechanisms. Metabolite analysis of the ASR interactions has confirmed some of these findings (Lygin et al., 2009). Several genomic studies involving TFs identified roles for WRKY, the Basic Leucine Zipper (bZIP) domain, and predicted TF families in resistant interactions (Pandey et al., 2011; Aoyagi et al., 2014; Bencke-Malato et al., 2014; Alves et al., 2015). The SuperSAGE technique was used to identify several antimicrobial peptides such as defensins, thionin, and lipid transfer protein (LTP) family genes in cowpea and soybean infected with the rust pathogen *P. pachyrhizi* (Kido et al., 2010).

Nonhost resistance (NHR) is a type of resistance that is displayed by plants against most potential pathogens. This type of resistance can be multi-layered, and plants can exhibit this either prior to infection (pre-invasive NHR) or post-infection (post-invasive NHR) (Senthil-Kumar and Mysore, 2013; Gill et al., 2015; Lee et al., 2017; Fonseca and Mysore, 2019). The NHR responses of Arabidopsis and *M. truncatula* against *P. pachyrhizi* were explored to identify sources of durable disease resistance. Studies with Arabidopsis have indicated that the ASR fungus exploits the necrotrophic pathway by inducing JA-mediated responses to evade host defense responses (Loehrer et al., 2008; Campe et al., 2014). The roles of *PENETRATION 1-4 (PEN1-4), SENESCENCE-ASSOCIATED GENE 101, BRIGHT TRICHOMES 1*, and *POSTINVASION-INDUCED NONHOST RESISTANCE GENES4/5/9* (*PING4/5/9*) in pre-invasive and post-invasive NHR mechanisms have been explored (Loehrer et al., 2008; Langenbach et al., 2013; Langenbach et al., 2016b). Furthermore, the potential of transferring NHR *PING* genes to soybeans and conferring enhanced ASR resistance has been demonstrated (Langenbach et al., 2016b). Transcriptional changes during the interaction of *P. pachyrhizi* with *M. truncatula* were used to identify genes involved in NHR (Ishiga et al., 2015). A combination of transcriptome and metabolite analysis indicated the role of the secondary metabolite, medicarpin, in inhibiting the germination and differentiation of rust urediniospores. Transcriptome analysis also indicated the role of chlorophyll catabolism genes in disease resistance. Further characterization of the *STAY GREEN* gene indicated its role in the hypersensitive-like response during the resistance interaction (Ishiga et al., 2015). Further, a forward genetics-based screening of *M. truncatula Tnt1* insertion lines (Tadege et al., 2008; Sun et al., 2019) for alterations in response against *P. pachyrhizi* identified the *inhibitior of rust germ tube differentiation1 (irg1)* mutant (Uppalapati et al., 2012). *IRG1* encodes a Cys(2)His(2) zinc finger transcription factor, PALM1, which plays a role in regulating epicuticular wax metabolism and transport, and epicuticular wax is important for ASR spore differentiation (Uppalapati et al., 2012; Ishiga et al., 2013).

An interesting study involving resistant interaction with two foliar pathogens, *Colletotrichum trifolii* (hemibiotrophic pathogen) and *Erysiphe pisi* (biotrophic pathogen) and a partially resistant root pathogen, *Phytophthora medicaginis* (necrotrophic pathogen), with *M. truncatula* identified three *Pathogenesis Related* (*PR*) 10 genes, a *TLP*, and a gene encoding hevein-like protein to be upregulated (Samac et al., 2011). The phenylpropanoid pathway involving isoflavonoid synthesis was also upregulated. Further characterization of these genes using RNAi lines identified the role of the *Chalcone Synthase* gene in the phenylpropanoid pathway in conferring resistance to necrotrophic pathogens. (Samac et al., 2011).

A quantitative PCR-based TF platform in *M. truncatula* was used to conduct TF expression profiling during interactions with *Uromyces striatus* (Madrid et al., 2010; Villegas-Fernández et al., 2014). The TF profiling in resistant interactions of *M. truncatula* with *U. striatus* identified genes encoding pathogenesis-related ethylene response factor (PR-ERF), WRKY, and the Myb class of TFs to be differentially expressed (Madrid et al., 2010). Comparing the TF expression profiling in the two pathosystems, *Botrytis* spp. and *U. striatus*, there were higher constitutively expressed TFs in *M. truncatula-Botrytis* spp. interactions, indicating an NHR-like response, although this resistance was compromised in the lab-experimental system, allowing infection. This may also be indicative of the differences in the host response to biotrophic versus necrotrophic pathogens (Madrid et al., 2010).

### Necrotrophic Pathogen Interactions

The availability of whole-genome microarrays of the model legume *M. truncatula* has advanced the understanding of various other fungal pathogen interactions in legumes (Uppalapati et al., 2009; Samac et al., 2011). *M. truncatula* is a susceptible host for Phymatotrichopsis root rot caused by the fungus *Phymatotrichopsis omnivora* (Uppalapati et al., 2010). Microarray analysis of this interaction identified JA- and ethylene-responsive genes, indicating a necrotrophic infection strategy (Uppalapati et al., 2009). Secondary metabolite genes involved in isoflavonoid synthesis were upregulated at the early infection stage but were eventually reduced to basal level during later disease progression stages, indicating the role of fungal manipulation of host defenses (Uppalapati et al., 2009).

Although *M. truncatula* is a nonhost for *Botrytis* spp., by screening several *M. truncatula* genotypes under lab conditions, a partially resistant genotype and a susceptible genotype were identified (Villegas-Fernández et al., 2014). This study identified *Botrytis fabae* as a more aggressive pathogen compared to *B. cinerea* on *M. truncatula*. However, microscopic studies indicate there is higher spore germination of the later pathogen species. Transcription factor (TF) profiling indicated that the host perceives *B. fabae* to be a more virulent pathogen by upregulating diverse TFs involved in stress responses even before the visible symptoms appear (Villegas-Fernández et al., 2014).

An integrated omics approach using RNAseq and metabolomics (1H NMR) data was used to understand the primary metabolism regulation of soybean in response to *Rhizoctonia solani* infection (Copley et al., 2017). A significant flux of responses in redox reactions and ROS signaling along with changes in peroxidases, post-infection, were observed in soybean leaves (Copley et al., 2017).

#### Genomics of Plant-Oomycete Interactions

The oomycete pathogens of legumes that have been most studied using genomic tools are *Phytophthora* spp and *Aphanomyces* spp. Some of the early studies of gene expression changes in soybean with *Phytophthora sojae* reflected the hemibiotrophic infection strategy of the pathogen at the molecular level (Moy et al., 2004). A cDNA microarray with genes from both host plant and pathogen was custom-built, and a time course of susceptible interaction studies revealed the expression of active defenses in the host mediated by SA-triggered pathways at early infection stages. This included expression of *PR1a* gene and genes involved in the phenylpropanoid pathway. The host and pathogen responses peaked at around 24 hpi, followed by a reduction in the host responses, indicating the shift from biotrophy to necrotrophy (Moy et al., 2004). With the availability of the Affymetrix^®^ gene chip for soybean, a detailed mapping of the soybean transcriptome change was carried out using three genotypes – resistant, partially resistant, and susceptible soybean varieties. This experiment was conducted with 72 biological replicates to understand the effect of genotypic variation on transcriptome changes (Zhou et al., 2009). The large number of replicates coupled with detailed statistical analysis demonstrated that almost the entire genome underwent low-level transcriptional changes in response to disease and genetic variation, yet most of the differences were less than two-fold in magnitude. This work hypothesized that these pervasive and statistically significant low-level changes may reflect the genotype-specific host adaptive changes in response to the pathogen and that studying these might be valuable. A macroarray study of resistant and susceptible interactions in the same disease system identified a role for putative regulators of chromosome condensation 1 protein family in the resistant interaction, suggesting the suppression of nucleocytoplasmic trafficking as one of the host strategies for combating disease (Narayanan et al., 2009). A more recent attempt to enrich the transcripts differentially expressed during the disease process employed the SSH cDNA library coupled with NGS (Xu et al., 2012). This study identified genes encoding several traditional proteins involved in disease-resistance strategies, including various PR-like proteins, the WRKY class of transcription factors, and proteins involved in the phenylpropanoid pathway. A novel discovery of this work involved identifying the allergen gene *Pru ar 1* (*Prunus armeniaca*) in soybean, which could be involved in resistance. Functional characterization of the *Pru ar 1* gene identified it as a novel gene encoding PR10 protein (Fan et al., 2015).

MicroRNAs (miRNAs) are 20- to 24-nucleotide long, single-stranded non-coding RNAs that play critical roles in various biological functions, including plant innate immunity. miRNAs-mRNA complexes regulate these responses (Navarro et al., 2008). Microarray analysis was conducted with susceptible, qualitative-resistant, and quantitative-resistant cultivars of soybean infected with *Phytophthora sojae* (Guo et al., 2011). This study identified different microRNAs in the three different interactions. The bioinformatics search indicated that some of the targets involved diverse categories such as defense response genes, kinases, transcriptional factors, etc. Several microRNAs have inverse expression patterns to their putative target genes. These data indicated a role for microRNAs in regulating plant defense responses in resistant interactions. To understand the single dominant gene in *Resistance to Phytophthora sojae* (*Rps*)-mediated resistance mechanisms in soybean-*P. sojae* interactions, researchers conducted transcriptome analysis of 10 near-isogenic lines, each with a unique *Rps* gene/allele (Lin et al., 2014). This study identified that *Rps* recognition was characterized by induction of SA-, ethylene-, and brassinosteroid phytohormone-signaling pathways, repression of JA pathways, ROS, WRKY transcription factors, MAP kinase-signaling pathways, and phytoalexin production. The compatible reaction was characterized by the induction of the JA pathway, repression of the ethylene pathway, and no changes to the SA and brassinosteroid pathways (Lin et al., 2014).

*Aphanomyces euteiches*, the causal organism of Aphanomyces root rot, is a major soilborne oomycete pathogen that infects various legume species, including pea, lentil, and alfalfa (Pilet-Nayel et al., 2009). In an attempt to understand the strategies employed during *A. euteuches* interactions with *M. truncatula* in a compatible interaction, a cDNA-AFLP approach was first employed to understand the optimal infection time for evaluation, followed by cDNA enrichment with SSH (Nyamsuren et al., 2003). This study identified classical *PR*- and defense genes. The molecular analysis indicated abscisic acid-mediated signaling that could induce PR-10 protein. The PR-4 protein-containing hevein domain, which could bind chitin, was also identified. A more recent study was conducted to compare the transcriptional responses in compatible interactions of pea plants with both the oomycete pathogens discussed here—*Phytophthora pisi* and *A. euteiches*—using a *M. truncatula* microarray (Hosseini et al., 2015). The study revealed different recognition and signaling components in the host against the two pathogens. PTI and ETI responses were detected in the early stages of infection with both pathogens. JA- and ET-hormone signaling were involved in both interactions, while the auxin-induced SAUR family proteins were specific to *A. euteiches*. The interactions of downy mildew pathogen, *Peranospora viciae* f. sp. *Pisi*, with pea leaves were investigated with SSH cDNA libraries (Feng et al., 2012). The study identified downy mildew resistance genes *RPP6/6/27* involved in this interaction.

#### Genomics of Plant–Bacteria Interactions

The application of genomic tools in understanding bacterial pathogenesis in legumes is relatively limited compared to in fungal pathogenesis studies. NGS technologies have been employed to understand the interactions of *Xanthomonas axonopodis* pv. *glycines* (*Xag*), which causes bacterial leaf pustule (BLP) disease in soybean (Kim et al., 2011; Chatnaparat et al., 2016). Kim et al. (2011) studied the transcriptome profiling in near-isogenic lines (NILs) of resistant and susceptible cultivars of BLP while Chatnaparat et al. (2016) studied the gene expression of the pathogen in susceptible host leaves. In the former study, several genes involved in PTI response such as *EF-TU RECEPTOR (EFR)*- and *FLAGELLIN SENSING 2 (FLS2)*-, *ATPASE 4 (ACA4)*-, *ACA11*-, *MAP KINASE 4 (MPK4)*-, *MPK6*-, and *RESPIRATORY BURST OXIDASE HOMOLOGUE (RBOH)*-like genes, and Damage-Associated Molecular Pattern (DAMP) receptors such as *PLASMA MEMBRANE LRR RECEPTOR KINASE 1(PEPR1)* and *PEPR2*, were induced at 0 hours post inoculation (hpi) in BLP-resistant NILs and not in BLP-susceptible NILs. Defense response genes such as *RPP*-, *RPM1*-, and *Mildew Locus O (MLO)*-like genes also were induced at this time point in BLP-resistant NILs. The authors speculate that this early up-regulation of PTI-related genes potentiates immune response during pathogen attack. Although the *Xanthomonas* species is known to be a biotroph, *Xag* behaves like a necrotrophic pathogen in soybean, as demonstrated by the molecular mechanisms in this study. Several genes encoding jasmonate-zim domain (JAZ)-like and MYC2 TF proteins were also induced at 0 hpi (Kim et al., 2011). This work demonstrates that the activity of PTI components at the early stage of infection is an important defense mechanism in the resistant soybean NIL tested.

Bacterial wilt caused by *Ralstonia solanacearum* is an important pathogen of peanuts, and genomic tools have been employed to understand the host–pathogen interactions. Early studies in this system were done using cDNA libraries where both the roots and leaves of peanuts were challenged with the pathogen, while in nature *Ralstonia* is a root pathogen (Huang et al., 2012). Ethylene and JA pathway genes were induced in both the roots and leaves of a highly resistant peanut cultivar. Several secondary metabolite genes were induced in roots and not in leaves, indicating the natural adaptation of the host to a root pathogen (Huang et al., 2012). In a more recent study of this pathosystem, NGS technology was used to study the gene expression differences between susceptible and resistant cultivars (Chen et al., 2014). In this study, the suppression of primary metabolism, especially carbohydrate metabolism, was an important feature in the resistant interaction, indicating the shift of energy investment from the primary metabolism to defense mechanisms. The PTI defense pathway was triggered in both resistant and susceptible interactions, and its partial suppression by the pathogen was observed. The expression patterns of secondary metabolites and defense response genes and hormone analysis indicated that resistance was primarily conferred by defense response genes in the ETI response cascade. Bacterial blight disease of soybean is caused by *Pseudomonas syringae* pv. *glycinea* (*Psg*). In a cDNA microarray study of both resistant and susceptible interactions of *Psg* with soybean using a virulent and avirulent strain of *Psg*, a three-phase response was studied. In phase I, which corresponded to the induction stage at 2 hpi, no significant differences were seen between susceptible and resistant interactions. Phase II, which lasted from 3 to 10 hpi, and phase III, up to 24 hpi, corresponded to the effector stage and programmed cell death (PCD) stages in resistant interaction, respectively. Several gene expression changes were observed in phases II and III. An important reported observation was a 92% reduction in the expression of chloroplast-related genes in the resistant interaction event at 8 hpi with no visible symptoms. Physiological measurements supported these data. There was a lack of ROS in the susceptible interaction at phase II. This study suggested the role of photosystem centers as a potential source of the secondary ROS or the oxidative burst response that eventually leads to PCD in phase III (Zou et al., 2005). *Pseudomonas syringae* pv. *syringae* causes bacterial stem blight in alfalfa. RNAseq was conducted to study the host–pathogen interactions in resistant and susceptible alfalfa cultivars at two different time points (Nemchinov et al., 2017). The timing of resistance response differed in both cultivars. The ZG9830 cultivar triggered ETI responses much earlier than the Maverick cultivar. The resistance response in cultivar ZG9830 may involve NBS-LRR, TIR-unknown (TX), and nematode-resistance proteins named based on their homology to Hs1^pro-1^ (*HSPRO2*)-like *R* genes, while the cultivar Maverick may involve the CNL class of *R* genes (Nemchinov et al., 2017).

#### Genomics of Nematode–Plant Interactions

*Heterodera glycines*, commonly known as soybean cyst nematode (SCN), is among the most devastating soybean pathogens. In an attempt to engineer resistance against SCN, detailed characterization of the molecular changes during the infection process in both soybean and the pathogen have been performed. cDNA-based microarrays were initially used to profile the transcriptome changes in the soybean roots during different infection stages in both compatible and incompatible interactions with SCN (Alkharouf et al., 2004; Khan et al., 2004; Alkharouf et al., 2006). These studies identified compatible interaction-specific and incompatible interaction-specific genes as well as time-based induction of genes. Stress-induced gene *PR-10* was identified in both compatible and incompatible interactions. Several genes belonging to carbohydrate metabolism, plant defense response, and signaling were indicated in compatible interaction. In a time-course study by Alkharouf et al. (2006), plant responses were documented prior to feeding cell selection (pre-FCS) as well as after feeding cell selection (post-FCS) during compatible interaction. The pre-FCS stages induced *PR-10* genes, stress-related genes, carbohydrate-metabolism genes, and secondary metabolism genes. The differentially expressed genes during post-FCS were involved in transcription and protein synthesis. Later studies employing the Affymetrix^®^ gene chip identified differentially expressed genes like *PR-5*, *PR1a*, *Expansins*, cell wall-fortification genes, and phenylpropanoid pathway genes during the post-FCS stage (Ithal et al., 2007). Differential gene expression changes were observed in different genotypes even during the pre-FCS based on the interaction type (compatible/incompatible) in whole-root analysis. Genes belonging to No Apical Meristem (NAM) domain-containing TFs, the WRKY class of TFs, Nucleotide Binding Site-Leucine Rich Repeat (NBS-LRR) kinases, signal transduction, cell wall-fortification, and GC-enriched elements in promoters of genes were identified in the incompatible interaction (Klink et al., 2007b; Mazarei et al., 2011; Wan et al., 2015). In an attempt to enrich for genes differentially expressed during pathogenesis, RNA isolated from laser-capture microdissection samples of syncytial cells was used for microarray analysis with the soybean Affymetrix^®^ gene chip to understand the defense responses in both compatible and incompatible interactions (Klink et al., 2007a; Klink et al., 2009b; Klink et al., 2010; Kandoth et al., 2011; Matsye et al., 2011). The developmental stages of syncytium are divided into a parasitism phase where the syncytium develops and a second phase when the resistance develops. Microarray studies of gene expression during these specific stages indicated that the whole-root analysis masked several key players that are involved in the specific interactions. There were no significant changes in gene expression in the parasitism stage in the compatible and incompatible interactions (Klink et al., 2010). *Lipoxygenases*, *14-3-3*, and genes involved in JA and ethylene biosynthesis, the S-adenosyl methionine pathway, the flavonoid pathway, and coumarin and cellulose biosynthesis were highly induced in the resistant interaction at different stages of resistance response (Klink et al., 2007a; Klink et al., 2009b; Klink et al., 2010). The gene expression profiling in *Resistance to H. glycines* (*Rhg1*)-mediated soybean resistance utilizing laser capture microdissections identified apoptosis-related, hypersensitive, and SA-induced defense response genes in the resistant interaction. Several of these genes were either partially or completely suppressed during susceptible interactions with SCN (Kandoth et al., 2011). Genotype-specific defense response studies in soybean indicated two different types of resistant responses involving the varieties Peking and PI88788. Resistance in the Peking variety involved rapid and potent cell wall appositions, while resistance in the PI88788 variety involved potent but slow response without cell wall appositions (Matsye et al., 2011). Microarray studies in these two varieties indicated the role of amino acid transporter and alpha soluble NSF (*N*-ethylmaleimide-sensitive factor) attachment protein in plant defense (Matsye et al., 2011). Another study with two different SCN populations that invoke a resistant and susceptible response in the same soybean genotype, Peking, revealed that SCN might have evolved different mechanisms to overcome host resistance (Klink et al., 2009a). Approximately 71 genes were induced and 44 genes were suppressed in the SCN strain that triggered a resistant reaction in the host during pre-infection stage. As the infection progressed, many SCN genes were suppressed in the resistant interaction. These data indicate that the feeding and nutritional uptake mechanisms of SCN might be the targets of the host defense. A recent study conducted by Tian et al. identified the microRNAs that are differentially expressed between two soybean cultivars, KS4313N and KS4607, which have differential resistance response to SCN. They identified a total of 60 differentially expressed miRNAs belonging to 25 families correlating to the response of the cultivars (Tian et al., 2017). Black soybean, Huipizhi Heidou, has different grades of resistance to SCN. RNAseq analyses at three different infection time points were conducted in two cultivars representing resistant and susceptible interactions (Li et al., 2018b). The study suggested roles for five plant hormones in the resistance. While SCN is a pathogen of soybean, it can also infect and reproduce in the roots of common bean, causing yield reductions. Gene expression profiling of common bean roots upon infection with SCN resulted in the differential expression of genes encoding nucleotide-binding site leucine-rich repeat resistance (NLR) proteins, WRKY TFs, PR proteins, and heat shock proteins (Jain et al., 2016).

*Meloidogyne* spp., commonly known as root-knot nematodes (RKN), are biotrophic parasites of soybean and cause major crop losses. The resistance mechanisms of incompatible soybean interactions with *Meloidogyne incognita* indicated auxin-mediated defense responses (Beneventi et al., 2013). Based on transcript profiling, a potential defense model was proposed in which ROS-mediated calcium signaling and nucleoside sugar formation play a critical role in plant hormone signaling. ROS homeostasis was proposed through a balance of auxin-mediated gibberellic acid (GA) and ROS-mediated JA and GA pathway signaling, which maintains low oxidative stress in plants and allows for plant growth. DELLA-like protein was proposed to be a key element in the plant hormone signaling pathway. On the other hand, the gene expression studies on the compatible interactions with RKN indicated an induction of genes involved in the cell cycle, sugar metabolism, and cell wall metabolism. These processes are involved in the successful establishment of giant cells during the infection process. Host defense response genes involving JA-mediated pathways were induced in the early infection stages, while most of them were downregulated by the time the infection had progressed, indicating that RKN actively manipulates host defenses (Ibrahim et al., 2011). The studies on the *Rk* locus-mediated resistant interaction of RKN indicated that most of the host defenses were suppressed during the infection and feedings stages. Based on this work, it was proposed that the host defenses are triggered against the nematode infection, likely due to the high accumulation of toxins involving unique resistance mechanisms (Das et al., 2010). NGS studies during the early and late stages of the compatible interaction of *M. incognita* in common bean identified biotic and abiotic stress responses (Santini et al., 2016). Enhanced expression of wound responsive genes at early stages and the TMV resistance protein encoding *N* gene indicated an active host response to block pathogen infection. This basal response was broken by suppression of ET/JA pathways and at later infection stage (Santini et al., 2016). *M. incognita* can also infect alfalfa and cause disease in some varieties or accessions. Resistant and susceptible interactions of *M. incognita* with alfalfa were profiled using both cDNA libraries and through NGS using Illumina Hiseq 2000 (Potenza et al., 2001; Postnikova et al., 2015). There was a high induction of defense-related and stress-response genes in susceptible interaction, indicating basal defense responses. Analysis with the bioinformatics platform for plant resistance (*R*) gene analysis, PRGdb, identified two potential resistance (*R*) genes specific to the resistant interaction. Recently, NGS was used to study the genomics of resistance in wild diploid peanut *Arachis stenosperma* that harbors resistance to *M. arenaria* (Guimaraes et al., 2015). This study identified components of genetic resistance and induced resistance that could be integrated into breeding programs for durable resistance to RKN.

#### Genomics of Plant–Virus Interactions

*Soybean mosaic virus* (SMV) is an RNA virus and is one of the most prevalent viral pathogens of soybean. A handful of genomics studies have been conducted to understand the molecular changes involved in this disease interaction, including transcriptomics, degradome-seq, and smallRNA-seq (sRNA-seq). One of the earliest genomic studies of SMV-soybean interaction was conducted using cDNA microarrays to investigate the transcriptional changes from early to late infection stages. This study revealed that the plant immune responses are activated at late infection stages in the compatible interaction and that this delayed defense response may be critical to establishing systemic infection (Babu et al., 2008). To study the impact of elevated ozone on the SMV–soybean compatible interaction, gene expression profiling was conducted using soybean microarrays. Increasing ozone concentrations delayed the onset of disease, and this delay corresponded to the expression of basal defense response genes (Bilgin et al., 2008). Comprehensive RNA-seq, sRNAseq, and degradome-seq were performed in soybean during compatible and incompatible interactions with SMV in two different studies (Chen et al., 2016; Chen et al., 2017). An miRNA-mRNA regulatory network was developed based on these data to elucidate the role of miRNAs in the SMV infection process. This study further identified 71 genes that potentially play a role in defense during SMV infection (Chen et al., 2016). One of the differentially expressed genes, *Eukaryotic Elongation Initiation Factor 5A* (*ElF5A*), was further characterized, and the knockout mutant of this gene was hyper-susceptible to SMV (Chen et al., 2017). A time-course RNA-seq study during the soybean–SMV compatible interaction identified roles for SA and NLR family genes that were downregulated during compatible interaction and upregulated during incompatible interactions (Zhao et al., 2018).

Transcriptional responses during *Bean common mosaic virus* (BCMV) interaction with common bean were investigated with two known and one unknown strains of BCMV. The known strains that caused moderate disease symptoms induced more transcriptional changes than the unknown strain that caused severe symptoms (Martin et al., 2016). More recently, a study was conducted to identify miRNAs during the infection of *Mungbean yellow mosaic India virus* (MYMIV) in common bean employing high-throughput sequencing and identified 107 differentially expressed miRNAs during infection and 3,367 potential target genes for these miRNAs (Patwa et al., 2018).

### Genomic Applications in Legume Breeding

#### Molecular Markers in Legume Plant–Pathogen Interactions

Engineering or breeding for resistance against plant diseases and nematodes is a more economical and eco-friendly approach than is the use of pesticides. The selective breeding process depends on the type of trait and whether the information for such resistance can be inherited in a qualitative or quantitative manner (Poland and Rutkoski, 2016). Qualitative disease-resistance breeding involves large screening assays that are often laborious and require extensive knowledge of plant–pathogen interactions. Lately, introgression of resistance genes into selective breeding material have relied on the use of molecular markers to assist breeders in the breeding process, which is often called Marker Assisted Selection (MAS) (Cobb et al., 2019). Molecular markers, including AFLPs, simple sequence repeats (SSRs), and more commonly single nucleotide polymorphisms (SNPs), have been developed in a variety of crops and used for different breeding programs. Nucleotide binding site (NBS) profiling, a new marker technology that improves the detection of molecular markers for disease resistance, was developed to identify markers by using NBS regions in the genomes (Van Der Linden et al., 2004). Due to its gene-targeting nature, NBS-profiling directs a PCR reaction to NBS domains through which a large number of *R* genes can be identified as molecular markers (Van Der Linden et al., 2004). More recently, the availability of genome sequence information for a number of plant species, including legumes, has helped the identification of molecular markers such as SNP markers that can be integrated into breeding programs for resistance screening. Some of the recent genomic resources available in legumes such as *M. truncatula*, *L. japonicus*, soybean, chickpea, and pigeon pea are described here (Sato et al., 2007; Sato et al., 2008; Schmutz et al., 2010; Young et al., 2011; Varshney et al., 2012; Varshney et al., 2013; Wang et al., 2013; Pecrix et al., 2018). Markers developed in a variety of ways are integrated into legume breeding programs for resistance against plant pathogenic fungi, oomycetes, bacteria, and nematodes. Though a common approach for engineering resistance into plants is through the integration of race-specific resistance against a known pathogen, this may not impart long or durable resistance, as the single *R* gene-mediated resistance can be overcome in an arms race by the rapidly evolving pathogens (Fonseca and Mysore, 2019). Hence, a better approach to create a durable resistance is through the deployment of quantitative trait loci (QTLs) through breeding strategies (Kou and Wang, 2010; Kou and Wang, 2012; Zhou et al., 2018) or through a transgenic approach using genes involved in NHR from different plant species (Fonseca and Mysore, 2019). Previous studies identified several genes involved in NHR against important legume pathogens (Fonseca and Mysore, 2019). *Phytophthora sojae* is a fungal pathogen that causes root rot in soybean and is non-pathogenic on *M. truncatula*, alfalfa, and Arabidopsis. Penetration mutants (*pen1-1*) in Arabidopsis were found to be compromised to *Phytophthora sojae*, thus establishing the case for pre-invasive non-host resistance (Sumit et al., 2012). This gene, when transferred to soybean, resulted in enhanced resistance to *Fusarium virguliforme* (Wang et al., 2018). Similarly, the Asian soybean rust pathogen, *P. pachyrhizi*, was not able to infect *M. truncatula*, alfalfa, or Arabidopsis (Loehrer et al., 2008; Langenbach et al., 2013; Ishiga et al., 2015). The strategy of transferring genes involved in NHR was used to increase the resistance of soybean to *P. pachyrhizi*. In this case study, 10 *PING* genes were overexpressed in soybean, resulting in enhanced resistance to *P. pachyrhizi* infections (Langenbach et al., 2016b).

In this section, we will explore the use of markers and QTLs in legume resistance breeding. Bulk segregant analysis (BSA) was used to map resistance to *A. euteiches* (*AER1*) in *M. truncatula* (Pilet-Nayel et al., 2009). Meta-QTL analysis in pea resulted in the identification of 27 meta-QTLs for resistance to *A. euteiches*. Six of them were found to co-localize with six of the meta-QTL regions identified for plant height and earliness (Hamon et al., 2013). Two major QTLs *Ae-Ps7.6* and *Ae-Ps4.5* were identified in pea near-isogenic lines (NILs) that were able to delay the symptoms of oomycete pathogen *A. euteiches* in pea (Lavaud et al., 2016).

Ascochyta blight (AB) of pea is caused by complex of fungal pathogens including *Didymella pinodes* and *Phoma medicaginis* var *pinodella*. QTLs of resistance to the blight complex pathogens were identified as QTLs based on two QTL mapping populations, A26 × Rovar and A88 × Rovar. QTL peaks, for the *Asc2.1*, *Asc4.2*, *Asc4.3*, and *Asc7.1* QTLs, were defined by four of the pea defense candidate genes (Timmerman-Vaughan et al., 2016). These regions were identified on linkage group I in the vicinity of markers c206 and sB17-655, on linkage group III in the vicinity of markers M2P5-169 and PI39, and on linkage group VII in the vicinity of markers Z12-2400, HSP18.1, and MAPKinase (Timmerman-Vaughan et al., 2016). Similarly, AB of dry pea is predominantly caused by *Didymella pisi*. In an effort to identify QTLs that show consistency across locations and years, Jha et al. (2016) identified two QTLs, *abIII-1* and *abI-IV-2*, for AB resistance. AB is also an important disease in faba bean, resulting in yield losses of 35-40% (Atienza et al., 2016). Two QTLs governing resistance to *Ascochyta fabae* were identified on chromosome II (Af2) and chromosome II (Af3) of faba bean (Atienza et al., 2016).

Rusts in pea are caused by pea rust pathogen *Uromyces pisi*. Using DArT-Seq and 8,514 SNP markers, two QTLs, *UpDSII* and *UpDSIV*, were identified in the Linkage Groups (LGs) II and IV that controlled resistance to *Uromyces pisi* (Barilli et al., 2018). In cowpea, rust is caused by the *Uromyces vignae* pathogen. A single dominant *R* gene (*Ruv2*) that confers resistance against *U. vignae* was found to be inherited in RILs against the *U. vignae* isolate, Auv-LS (Wu et al., 2018).

Powdery mildew in pea is caused by *Erysiphe pisi*. The infection results in the formation of small diffused spots on the upper surface of the leaves and at advanced stages covers the entire plant surfaces as a white powdery growth (Ek et al., 2005). Powdery mildew resistance in pea is governed by a pair of recessive alleles “*er1er1*” (W.H, 1948; Tiwari et al., 1997). Humphry et al. (2011) identified the *Er1* locus as *PsMLO1* and established through complementation that the loss of *PsMLO1* function conditions durable broad-spectrum powdery mildew resistance in pea. Besides the recessive allele *er1*, another recessive allele *er2* and a dominant gene *Er3* was recently identified and reviewed by Fondevilla and Rubiales (Fondevilla and Rubiales, 2012). *Er3*, the dominant gene conferring resistance to powdery mildew in pea, was mapped to pea linkage group IV (Cobos et al., 2018). A variety of molecular markers closer to the *Er* locus were developed to screen the genotypes of pea for powdery mildew resistance (Ek et al., 2005; Sun et al., 2016a; Ganopoulos et al., 2018). A novel *er1-7* allele conferring pea powdery mildew resistance was identified through a 10-bp deletion in *PsMLO1* cDNA (Sun et al., 2016b). Other natural variations of *er1* alleles have been identified, and markers have been designed to screen for powdery mildew resistance in pea (Sudheesh et al., 2015; Sun et al., 2016a; Sun et al., 2016b).

Pea root rot is caused by a variety of fungal plant pathogens, and the causal agent has been identified as *Fusarium solani fsp. pisi* (*Fsp*). A strong QTL, *Fsp-Ps 2.1*, governing resistance to *Fsp* has been detected in the recombinant inbred line (RIL) populations of pea (Baccara × PI 180693). The QTL *Fsp-Ps 2.1* has been identified along with two other minor variance QTLs using three criteria: root disease severity, ratios of diseased vs. healthy shoot heights, and dry plant weights under controlled conditions (Coyne et al., 2019).

Soybean cultivation is significantly affected by SCN and by the sudden death syndrome (SDS) caused by the soilborne fungus *F. virguliforme*. SDS of soybean results in necrosis/rot of roots, while SCN infection results in yellow dwarf symptoms in soybean. Using soybean plant populations resistant to SCN and SDS, QTL mapping populations have been developed to identify QTLs for both SDS and SCN (Swaminathan et al., 2018).

Verticillium wilt, caused by the soil borne fungus *Verticillium alfalfae*, is one of the most serious diseases of alfalfa. Through the use of BSA on the alfalfa genotypes and by using marker-trait associations with the help of SSRs and SNPs, 17 SNP markers linked to Verticillium wilt resistance were identified (Zhang et al., 2014). Similarly, using *M. truncatula* as a model to develop QTLs for resistance against Verticillium, a population of recombinant inbred lines (RILs) from a cross between resistant line F83005.5 and susceptible line A17 were inoculated with a potato isolate of *V. albo-atrum*, LPP0323. Following the inoculation and screening, a set of four QTLs were identified for the area under the disease progress curve and for maximum symptom score (Negahi et al., 2014). A similar study design was used to identify three distinct QTLs (MtVa1, MtVa2 and MtVa3) that confer resistance to *V. albo-atrum* in a population of A17 and DZA45.5 (Ben et al., 2013). A recent transcriptomic study conducted on the early root responses of *M. truncatula* lines A17 (resistant) and a susceptible line (F83005.5) identified core transcriptional responses against root pathogens and showed that the resistance line A17 displayed higher defense-related genes upon inoculation with *V. alfalfae* V31-2 (Toueni et al., 2016). Phytophthora root rot is caused by an oomycete pathogen *Phytophthora sojae*, resulting in damping-off, yellowing and wilting diseases in soybean (Li et al., 2017). A QTL, *Resistance to Phytophthora sojae* (*RpsQ*), which confers resistance against *P. sojae* in soybean cultivar Qichadou 1, was mapped using SSR markers to a 118-kb region on the soybean chromosome 3. This 118-kb mapped region consists of 11 candidate genes, and one of them, *Glyma.03g027200*, was found to encode a serine threonine receptor-like kinase (RLK), which was later confirmed as a likely candidate gene of *RpsQ*. In a study of *M. truncatula* roots colonized by pathogenic oomycete *Phytophthora palmivora*, SNP markers associated with plant colonization response were identified upstream of a *Required for Arbuscule Development 1 (RAD1)* locus, a positive regulator of arbuscular mycorrhizal (AM) fungus (Rey et al., 2017). The *rad1* mutant was impaired in colonization by AM fungi as well as by *P. palmivora* (Rey et al., 2017). This is one example showing how the use of association mapping in legumes can help identify the genes responsible for genetic resistance against an oomycete pathogen. Readers are encouraged to read the more recent review on fungal root diseases in grain legumes and the implications of plant genetic variation in plant breeding (Wille et al., 2019).

Anthracnose of lentils is caused by *Colletotrichum lentis* and accounts for 70% of the crop loss in lentils (Bhadauria et al., 2019). Recent genomic sequencing studies on one of the pathogenic races of *C. lentis* (virulent race 0) combined with QTL mapping led to the identification of a single QTL, *qClVIR-11*, located on mini chromosome 11, thus explaining 85% of the variability in virulence of the *C. lentis* population (Bhadauria et al., 2019).

Cowpea is one of the highly cultivated legume crops and is susceptible to many biotic stresses caused by nematodes, bacteria, and fungi. Root-knot nematodes (RKN) are the most important pests of cowpea, resulting in huge losses due to their interference with the root architecture, which results in poor development of the plants (Santos et al., 2018). Previously, two resistance genes, *Resistance to Root knot* (*Rk*) and *Rk* *^2^*, were identified to confer resistance against RKN in cowpea (Das et al., 2010; Ndeve et al., 2019). A recent QTL mapping study using RIL population 524B x IT84S-2049 in cowpea resulted in the identification of a major QTL, *QRk-vu9.1*, associated with resistance to *Meloidogyne javanica* reproduction (Santos et al., 2018). This QTL was mapped on linkage group LG9 at position 13.37 cM using egg production data. Interestingly, the mapped intervals for this QTL corresponded with six *TIR-NBS-LRR* (*TNL*) genes that were identified using transcriptomic analysis between NILs resistant and susceptible to RKN (Santos et al., 2018). A majority of the examples quoted here are in early studies towards achieving economic benefits by developing disease-resistant cultivars.

#### Use of GWAS in Legume–Pathogen Interactions

In plant species, underlying variation with phenotypic data can be quantified, and genome-wide association mapping (GWAS) can be applied for identifying genes and for associating them with the phenotypes. This type of GWAS analysis for SNP discovery is made possible through the development of several target-enrichment or reduction-of-genome-complexity methods such as Genotyping-by-Sequencing (GBS) (Elshire et al., 2011; Glaubitz et al., 2014) or restriction site-associated DNA sequencing (RADSeq) (Davey and Blaxter, 2010). RADseq combines two simple molecular biology methods such as restriction digestion of DNA into fragments and then tagging them using identifier tags followed by NGS (Das et al., 2010). Recently, Diversity Arrays Technology (DArT) in combination with NGS platforms, known as DArTseq™, was used to develop a relatively large number of polymorphic markers to build dense genetic maps (Kilian et al., 2012; Kilian and Graner, 2012). In the GBS methodology, genome complexity is reduced through the use of methylation-sensitive restriction enzymes. Such a method helps avoid the sequencing of repetitive regions and can aid in the sequencing of low copy regions with high efficiency (Elshire et al., 2011). The majority of the GWAS studies described below in this section followed the GBS methodology to generate the genotyping data for SNP identification and exclusively used the GWAS pipelines developed for model and non-model plants (Lipka et al., 2012; Glaubitz et al., 2014; Tang et al., 2016). A variety of experiments have been conducted using GWAS to study the variations associated with the phenotypes in Arabidopsis (Atwell et al., 2010), Maize (Tian et al., 2011), rice (Huang et al., 2010), soybean (Lam et al., 2010), and *M. truncatula* (Branca et al., 2011). In this section, we will review GWAS studies of legumes in relation to disease resistance phenotypes. Alternatively, HapMap accessions that are sequenced by whole genome sequencing provide an excellent opportunity for the identification of SNPs across the HapMap populations (http://www.medicagohapmap.org/). Some of the *M. truncatula* HapMap accessions (288 accessions) were used to map flowering time traits linked to nitrogen fixation and for identifying other traits of interest (Stanton-Geddes et al., 2013; Kang et al., 2015; Kang et al., 2019). GWAS was used to estimate linkage disequilibrium levels and identify quantitative resistance loci (QRL) controlling resistance to both anthracnose and Angular leaf spot (ALS) diseases of 180 accessions of common bean. The study resulted in the identification of 21 and 17 statistically significant SNPs associated with anthracnose and ALS diseases of common bean, respectively (Perseguini et al., 2016). Bonhomme and his colleagues (Bonhomme et al., 2014) used high-density SNPs (∼5.1 million single nucleotide polymorphisms) to perform GWAS studies with Aphanomyces root rot resistance against 179 HapMap accessions of *M. truncatula*. With the use of GWAS, they were able to identify two QTL loci on chromosome 3, with candidate SNPs in the promoter and coding regions of an F-box protein coding gene (Bonhomme et al., 2014). GWAS was recently used in 175 *Pisum sativum* lines and were genotyped for resistance to *A. euteiches* using 13,204 SNPs from the GenoPea Infinium^®^ BeadChip (Desgroux et al., 2016). The study resulted in the identification of 52 QTLs of small size intervals associated with resistance to *A. euteiches* and further validated six of the seven previously reported QTLs (Desgroux et al., 2016). A similar GWAS study was performed to find the association between the plant system architecture of pea and *A. euteiches* resistance by using 266 pea lines that varied in both of the traits (plant system architecture and disease resistance). Genotyping the lines with 14,157 SNP markers resulted in the identification of one significant SNP mapped to major QTL *Ae-Ps7.6* associated with both resistance and root system architecture (RSA) traits (Desgroux et al., 2017).

Brown stem rot (BSR) of soybean, caused by the soilborne fungus *Cadophora gregata*, affects soybean production in the Northern United States, Canada, and Brazil. Using GWAS, a BSR resistance QTL has been identified in chromosome 16 and is located between 32.8 and 33.1 Mb based on the Glyma2.0 assembly (Rincker et al., 2016). More importantly, this region also maps to previously identified *Resistance to Brown Stem Rot* genes (*Rbs*) *Rbs1*, *Rbs2*, and *Rbs3* (Rincker et al., 2016). This narrow range of resistance QTL could be useful for MAS breeding programs. A comprehensive global view of disease resistance loci in soybean against multiple plant pathogens was presented through the use of GWAS on public Germplasm Resources Information Network and public SNP data (SoySNP50K; (Chang et al., 2016). Using GWAS, the authors identified significant novel SNPs associated with resistance to: bacterial pustule caused by *Xanthomonas axonopodis* pv. *glycines*; BSR caused by fungus *C. gregata*; Diaporthe stem canker caused by *Diaporthe phaseolorum* var. *caulivora* and *D. phaseolorum* var. *meridionalis*; SDS caused by *F. virguliforme*; ASR caused by *P. pachyrhizi*; SCN caused by reniform nematode, *Rotylenchulus reniformis*; and bean pod mottle virus (Chang et al., 2016).

GWAS was applied to detect SNPs significantly associated with resistance to *H. glycines* in the core collection of the common bean. There were 84,416 SNPs identified in 363 common bean accessions (Wen et al., 2019). GWAS identified SNPs on chromosome 1 that were significantly associated with resistance to *H. glycines* type 2.5.7. These SNPs were in linkage disequilibrium with a gene cluster orthologous to the three genes at the *Resistance to H. glycines* (*Rhg1*) locus in soybean. A novel signal on chromosome 7 was detected and associated with resistance to *H. glycines* type 1.2.3.5.6.7. Genomic predictions for resistance to these two *H. glycines* types in common bean achieved prediction accuracy of 0.52 and 0.41, respectively (Wen et al., 2019).

The molecular markers developed in legume species such as *M. truncatula*, pea, lentil, faba bean, and lupin can be used in other legumes. Recently, the transferability of molecular markers was tested in legumes such as chickling pea (*Lathyrus cicera*) and grass pea (*L. sativus*) (Almeida et al., 2014). During this study, ∼130 markers were successfully cross-amplified in *L. cicera* and *L. sativus* with an efficiency of 55% for gene-based markers (Almeida et al., 2014). Such comparative mapping can greatly boost the use of resources and expand the knowledge base in other related species as well.

#### Gene Editing in Legume–Pathogen Interactions

Gene introgression through breeding often comes with some undesirable trait inheritance that can perturb a desired outcome. Hence, the integration of plant breeding with precise editing of target genes can efficiently aid in the implementation of pathogen resistance in plants. This precision editing is recently made possible through the use of programmable sequence-specific nucleases such as zinc finger nucleases (ZFNs), transcription activator-like effector nucleases (TALENs), and, more recently, the clustered regularly interspaced short palindromic repeat (CRISPR)-associated protein9 (Cas9)-based genome editing tool (CRISPR/Cas9). These tools effectively generate target site mutations based on base-pairing of the engineered single-guide RNAs (sgRNAs) to the target DNA sites. More information on the development and applications of the CRISPR-Cas9 technologies in plant genomes can be found in previous reviews (Cong et al., 2013; Jiang et al., 2013; Perez-Pinera et al., 2013; Shan et al., 2013; Belhaj et al., 2015; Piatek et al., 2015; Kleinstiver et al., 2016; Ma et al., 2016; Tsai and Joung, 2016; Knott and Doudna, 2018). CRISPR/Cas9 or TALEN entry vectors were developed for gateway cloning in soybean and *M. truncatula* (Curtin et al., 2018). Several new web tools, such as E-CRISP (Heigwer et al., 2014) and CHOPCHOP (Montague et al., 2014), for the identification of CRISPR-Cas9 target sites are available both for target site identification and also for identification of off-target sites. In legumes, one such tool was developed for CRISPR/Cas9 design (Michno et al., 2015), and a methodology to perform gene editing in *M. truncatula* also is available (Curtin, 2018).

Using the CRISPR/cas9 technology, severe loss-of-function mutants were developed in the necrotrophic fungal pathogen *Sclerotinia sclerotiorum*. Using the previously characterized *Ssoah1* gene as the gene target, insertional gene mutants were generated that were found to be less virulent on soybean, *Brassica* spp. and tomato (Li et al., 2018a). Similarly, gene editing was adapted in *P. sojae* to generate mutants of *P. sojae* by manipulating *Avr4/6* genes of the pathogen (Fang and Tyler, 2016). These studies were important for determining the function of fungal or oomycete genes in pathogen virulence.

Recently, it has been demonstrated that, by using CRISPR/Cas9 genome editing of promoters, diverse cis-regulatory alleles can be generated and that quantitative variation can be an invaluable tool for breeding. A genetic scheme designed by Rodriguez-Leal et al. (2017) exploits transgenerational heritability of Cas9 activity in heterozygous loss-of-function mutant backgrounds. Such a system could also be used in the screening of QTLs for disease resistance if we knew the functions of the cis-regulatory alleles and could be a valuable tool for breeding (Rodriguez-Leal et al., 2017). This concept of generating variations was made possible through the use of epimutagenesis, a method that rapidly generates DNA methylation variation through random demethylation. This ability to manipulate plant methylomes to create epigenetically distinct individuals could be an invaluable breeding tool (Ji et al., 2018). Even though currently not many legume plants have been gene-edited to confer resistance against pathogens, in the future, we anticipate that gene editing will be used more frequently to engineer legume plants with yield-saving disease resistance.

## Conclusion

The advancements in cost-economic sequencing technologies have enabled global transcription profiling during plant–pathogen interactions in legumes and identified several pathways and candidate genes responsible for either disease susceptibility or resistance (**Table 1**). This progress has enabled a broader understanding of both plant and pathogen strategies during resistant and susceptible disease interactions. These studies have identified a repertoire of candidate genes that play key roles in resistance or disease processes. However, functional studies to evaluate their roles in plant–pathogen interactions are limited in some legume species, largely due to lack of mutant resources and appropriate methods for gene function validation. The *Tnt1*-mediated insertion mutagenesis in *M. truncatula* has generated ∼21,000 lines with ∼90% gene-tagging coverage in the genome (Tadege et al., 2008; Cheng et al., 2014; Sun et al., 2019). This genetic resource has been utilized to evaluate some candidate genes involved in plant–pathogen interactions. Similarly, several genetic resources are being developed for other legume species such as soybean and *L. japonicus* (Sato et al., 2007; Libault and Dickstein, 2014). Functional characterization of a few candidate genes has been achieved through RNAi methods and recombinant gene expression studies (Singh et al., 2013). Several genes identified in microarray analysis of SCN–soybean interactions have been characterized by overexpression studies and grouped into genes that enhance, reduce, or have no impact on disease susceptibility (Matthews et al., 2013). Such studies will augment the genomics data generated through whole-genome transcriptional studies. A variety of molecular markers, including AFLPs, SSRs, and SNPs, have been developed and then used to identify QTLs governing resistance to fungal and bacterial pathogens and to root-knot nematodes (**Table 2**). More recently, the underlying phenotypic variations combined with genotype information (SNPs) have been used for GWAS and are being used extensively in legume crops to identify the QTLs associated with the resistance loci against plant microbes. Precise genome editing technologies such as CRISPR-Cas9 have been employed to effectively knock out *P. sojae* effector *Avr4/6* and uncover the functional role of the corresponding resistance gene *RPS4/6* (Fang and Tyler, 2016). The utilization of these resources will help the biological function of genes identified through various genomic approaches to be better understood. Introgression of plant defense-related traits identified through genomics is in its early infancy and could lead to an economic success in the next few years. We predict that the use of the genomics tools in breeding mentioned in this review such as the use QTL introgression, GWAS, and CRISPR/cas9 editing of the genomes for generating plant variation will become increasingly popular in the next few years and will further advance our understanding as well as define our approaches to making improved cultivars in legumes. Genomic studies of plant–pathogen interaction will continue to provide us with novel disease resistance or defense-related genes that can be incorporated into elite legume cultivars, either by classical breeding or by biotechnological approaches.

**Table 1:** Summary of genomic methods and legume–pathogen interaction studies.

Plant	Pathogen	Methods	References
***Legume–fungus Interactions***			
Common bean	*Fusarium oxysporum* f. sp. *phaseoli*	cDNA-AFLP	Xue et al., 2015
Soybean	*Fusarium oxysporum* Schltdl	NGS	Lanubile et al., 2015; Chang et al., 2019
Chickpea	*Fusarium oxysporum* f. sp. *ciceri*	EST analysis	Ashraf et al., 2009
Chickpea	*Fusarium oxysporum* f. sp. *ciceri*	cDNA-AFLP	Nimbalkar et al., 2006; Gupta et al., 2009; Gupta et al., 2010
Chickpea	*Fusarium oxysporum* f. sp. *ciceri*	cDNA-RAPD	Nimbalkar et al., 2006; Gurjar et al., 2012
Chickpea	*Fusarium oxysporum* f. sp. *ciceri*	Long-SAGE	Upasani et al., 2017
Chickpea	*Fusarium oxysporum* f. sp. *ciceri*	NGS	Gupta et al., 2017
Chickpea	*Fusarium oxysporum* f. sp. *ciceri*	Microarray	Ashraf et al., 2018
*M. truncatula*	*Phymatotrichopsis omnivora*	Microarray	Uppalapati et al., 2009
*M. truncatula*	*Botrytis fabae*	qPCR-based transcription factor platform analysis	Villegas-Fernández et al., 2014
Soybean	*Rhizoctonia solani*	NGS	Copley et al., 2017
*M. truncatula*	*Mycosphaerella pinodes*	Microarray	Fondevilla et al., 2011
Pea	*Mycosphaerella pinodes*	DeepSuperSAGE	Fondevilla et al., 2014
Grass pea	*Ascochyta lathyri*	DeepSuperSAGE	Almeida et al., 2015
Fava bean	*Ascochyta fabae*	DeepSuperSAGE	Madrid et al., 2013
Fava bean	*Ascochyta fabae*	*De novo* transcriptome	Ocaña et al., 2015
Cowpea	*Phakopsora pachyrhizi*	SuperSAGE/DeepSuperSAGE - NGS	
*M. truncatula*	*Colletotrichum trifolii, C. lindemuthianum, C. higginsianum*	Microarray	Jaulneau et al., 2010
Common bean	*Colletotrichum lindemuthianum*	EST analysis	Oblessuc et al., 2012
Common bean	*Colletotrichum lindemuthianum*	NGS	Padder et al., 2016
Lentil	*Colletotrichum lentis*		Bhadauria et al., 2017
Soybean	*Phakospora pachyrhizi*	SSH-cDNA	Choi et al., 2008
*Glycine tomentella*	*Phakospora pachyrhizi*	Microarray	Soria-Guerra et al., 2010a; Soria-Guerra et al., 2010b
Soybean	*Phakospora pachyrhizi*	Microarray	Van De Mortel et al., 2007; Panthee et al., 2009; Morales et al., 2013
Soybean	*Phakopsora pachyrhizi*	SuperSAGE	Kido et al., 2010
*M. truncatula*	*Phakospora pachyrhizi*	Microarray	Ishiga et al., 2015
*M. truncatula*	*Colletotrichum trifolii, Erysiphe pisi, Phytophthora medicaginis*	Microarray	Samac et al., 2011
*M. truncatula*	*Uromyces striatus*	qPCR-based transcription factor platform	Madrid et al., 2010
***Legume–oomycete interactions***			
Soybean	*Phytophthora sojae*	cDNA microarray	Moy et al., 2004
Soybean	*Phytophthora sojae*	Comparative EST analysis	
Soybean	*Phytophthora sojae*	Microarray	Zhou et al., 2009
Soybean	*Phytophthora sojae*	SSH-cDNA - NGS,	Xu et al., 2012
Soybean	*Phytophthora sojae*	dot blot hybridizations	
Soybean	*Phytophthora sojae*	Subtractive EST analysis	Narayanan et al., 2009
Soybean	*Phytophthora sojae*	Microarray	Guo et al., 2011; Lin et al., 2014
			
*M. truncatula*	*Aphanomyces euteiches*	cDNA - AFLP, cDNA - SSH	Nyamsuren et al., 2003
Pea	*Aphanomyces euteiches; Phytophthora pisi*	Microarray	Hosseini et al., 2015
Pea	*Peronospora viciae* f. sp. *pisi*	SSH-cDNA	Feng et al., 2012
***Legume–bacteria interactions***			
Soybean	*Xanthomonas axonopodis* pv. *glycines*	NGS	Kim et al., 2011
Peanut	*Ralstonia solanacearum*	cDNA library analysis	Huang et al., 2012
Peanut	*Ralstonia solanacearum*	NGS	Chen et al., 2014a
Soybean	*Pseudomonas syringae* pv. *glycinea*	cDNA microarray	Zou et al., 2005
*M. sativa*	*Pseudomonas syringae* pv. *syringae*	NGS	Nemchinov et al., 2017
***Legume–nematode interactions***			
Soybean	*Heterodera glycines*	cDNA microarray	Alkharouf et al., 2004 and Khan et al., 2004; Alkharouf et al., 2006
Soybean	*Heterodera glycines*	Microarray	Ithal et al., 2007; Klink et al., 2007a; Klink et al., 2009a; Klink et al., 2009b; Klink et al., 2010; Kandoth et al., 2011; Matsye et al., 2011
Soybean	*Heterodera glycines*	NGS	Tian et al., 2017
Black soybean	*Heterodera glycines*	NGS	Li et al., 2017
Pinto bean	*Heterodera glycines*	NGS	Jain et al., 2016
Soybean	*Meloidogyne incognita*	NGS	Beneventi et al., 2013
Soybean	*Meloidogyne incognita*	Microarray	Ibrahim et al., 2011
*M. sativa*	*Meloidogyne incognita*	cDNA libraries	Potenza et al., 2001
*M. sativa*	*Meloidogyne incognita*	NGS	Postnikova et al., 2015
Bean	*Meloidogyne incognita*	NGS	Santini et al., 2016
Wild peanut	*Meloidogyne arenaria*	NGS	Guimaraes et al., 2015
			
***Legume-virus interactions***			
Soybean	*Soybean mosaic virus*	cDNA microarray	Babu et al., 2008
Soybean	*Soybean mosaic virus*	Microarray	Bilgin et al., 2008
Soybean	*Soybean mosaic virus*	NGS	Chen et al., 2016; Chen et al., 2017; Zhou et al., 2018
Common bean	*Mungbean yellow mosaic India virus*	High-throughput sequencing	Patwa et al., 2018

**Table 2:** Summary of QTL/marker analysis in legume–pathogen interaction studies.

Plant	Pathogen	Method	QTL/R-gene	Reference
*M.truncatula*	*Aphanomyces euteiches*	BSA	*AER1+D2:D25*	Pilet-Nayel et al., 2009
*M.truncatula*	*Aphanomyces euteiches*	GWAS using 5.1 million high-density SNPs	2 QTLs on chr3	Bonhomme et al., 2014
Pea	*Aphanomyces euteiches*	Meta-QTL analysis	*AER1*	Hamon et al., 2013
Pea	*Aphanomyces euteiches*	QTL mapping	*Ae-Ps7.6* and *Ae-Ps4.5*	Lavaud et al., 2015; Lavaud et al., 2016
Pea	*Aphanomyces euteiches*	GenoPea Infinium® BeadChip	*52 QTLs*	Desgroux et al., 2016
Pea	*Aphanomyces euteiches*	GWAS	*Ae-Ps7.6*	Desgroux et al., 2017
Faba bean	*Ascochyta fabae*	QTL mapping	*Af2* and *Af3*	Atienza et al., 2016
Pea	*Ascochyta pisi*	QTL mapping	*Asc2.1*, *Asc4.2*, *Asc4.3* and *Asc7.1*	Timmerman-Vaughan et al., 2016
Pea	*Ascochyta pisi*	QTL mapping	*abIII-1* and *abI-IV-2*	Jha et al., 2016; Jha et al., 2017
Chickpea	*Ascochyta rabiei*	WGS	*AB4.1*	Li et al., 2017
Soybean	*Cadophora gregata*	GWAS	*Rbs*	Rincker et al., 2016
Common bean	*Colletotrichum lindemuthianum, Pseudocercospora griseola*	GWAS	21 and 17 significant SNPs	Perseguini et al., 2016
Soybean	*Diaporthe phaseolorum, Cadophora gregata, Xanthomonas axonopodis pv. glycines*	GWAS using SoySNP50k	NovelSNPs	Chang et al., 2016
Pea	*Erysiphe pisi*	Deletion mapping	*er1*	Sun et al., 2016a; Sun et al., 2016b; Ganopoulos et al., 2018
Pea	*Fusarium solani fsp. pisi (Fsp)*	QTL mapping	*Fsp-Ps 2.1*	Coyne et al., 2019
Soybean	*Heterodera glycines*	QTL mapping	Novel QTL	Wen et al., 2019
Cowpea	*Meloidogyne incognita*	QTL mapping	*Rk and Rk2*	Das et al., 2010; Ndeve et al., 2019
Cowpea	*Meloidogyne javanica*	QTL mapping	*QRk-vu9.1*	Santos et al., 2018
*M.truncatula*	*Phytophthora palmivora*	Association mapping	*RAD1*	Rey et al., 2017
Soybean	*Phytophthora sojae*	SSR markers	*RpsQ*	Li et al., 2017
Pea	*Uromyces pisi*	DArT-Seq	*UpDSII* and *UpDSIV*	Barilli et al., 2018
Cowpea	*Uromyces vignae*	BSA	*Auv-LS*	Wu et al., 2018
*M.truncatula*	*Verticillium albo-atrum*	QTL mapping	*MtVa1, MtVa2 and MtVa3*	Ben et al., 2013
Alfalfa	*Verticillium alfalfae*	BSA	*17 SNPs*	Zhang et al., 2014

## Author Contributions

PK and KM conceived the idea. PK and RN wrote the manuscript. KM edited the manuscript.

## Funding

Projects in the KSM laboratory are funded by the Noble Research Institute, LLC, and the National Science Foundation. We thank Courtney Leeper (Noble Research Institute) for editing the manuscript.

## Conflict of Interest

The authors declare that the research was conducted in the absence of any commercial or financial relationships that could be construed as a potential conflict of interest.
